# Activated excited-state isomerisation in a hemipiperazine photoswitch

**DOI:** 10.1039/d6cc05789f

**Published:** 2026-09-28

**Authors:** Eleanor K. Ashworth, Anam Fatima, Samuel Bleckmann, Zbigniew L. Pianowski, Joseph A. Wright, Stephen R. Meech, Partha Malakar, James N. Bull

**Affiliations:** a Chemistry, Faculty of Science, University of East Anglia Norwich NR4 7TJ United Kingdom james.bull@uea.ac.uk; b Institute of Organic Chemistry (IOC), Karlsruhe Institute of Technology 76131 Karlsruhe Germany; c Institute of Biological and Chemical Systems-Functional Molecular Systems IBCS-FMS, Karlsruhe Institute of Technology Karlsruhe Germany; d International Institute of Molecular Mechanisms and Machines, Polish Academy of Sciences IMol PAN Warszawa Poland; e Central Laser Facility, Research Complex at Harwell, Rutherford Appleton Laboratory Didcot OX11 0QX United Kingdom

## Abstract

Hemipiperazines are an emerging class of robust molecular photoswitches based on a 2,5-diketopiperazine scaffold. We resolve the excited-state pathway responsible for efficient photoisomerisation of a model hydrogen-bonded hemipiperazine photoswitch using ultrafast time-resolved infrared spectroscopy and temperature-dependent fluorescence measurements.

## Abstract 2

Hemipiperazines are an emerging class of robust molecular photoswitches based on a 2,5-diketopiperazine scaffold.

## Graphical abstract

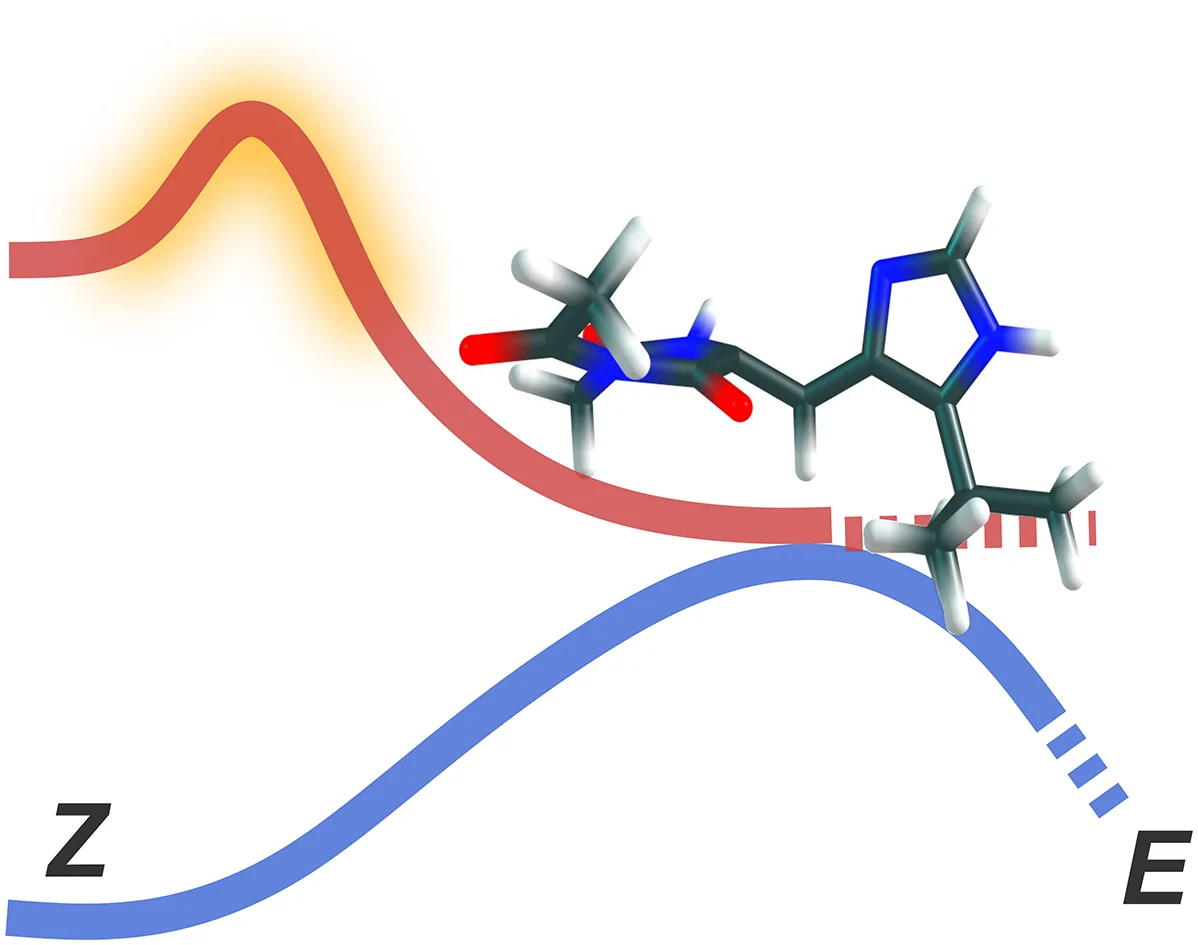

Unsaturated 2,5-diketopiperazines (DKPs) have long been known to exhibit geometrical isomerism and, in isolated cases, light-induced interconversion,^1,2^ but their systematic development as molecular photoswitches is recent. Pianowski and co-workers established 3-arylidene-2,5-diketopiperazines as a distinct class of reversible photoswitch, termed hemipiperazines, following the observation of photochromism in the antimitotic agent plinabulin,^3^ later applied to photomodulate development of zebrafish embryos.^4^ Subsequent studies have demonstrated extensive diversification of the hemipiperazine scaffold through variation of the arylidene substituent and extension or annulation of the conjugated framework.^5–7^

Of interest here is the imidazole-containing hemipiperazine, HP (Fig. 1a), previously reported as a synthetic precursor to plinabulin.^3^ The imidazole substituent supports an intramolecular hydrogen bond with the diketopiperazine framework, constraining the conjugated chromophore and placing the hydrogen-bonding interaction across the photoisomerising C=C bond. HP undergoes reversible *Z* ↔ *E* photoisomerisation,^3^ but the excited-state pathway through which this switching occurs has not been established. This comparatively simple member of the hemipiperazine family therefore provides a useful model for determining how the intrinsic excited-state potential-energy landscape and local hydrogen-bonding environment govern photoisomerisation in this class of hemipiperazines. Here, we investigate the excited-state relaxation and *Z* → *E* photoisomerisation dynamics of the synthesised *Z* isomer (lowest energy form) of HP using ultrafast time-resolved infrared (TR-IR) spectroscopy and temperature-dependent fluorescence measurements, identifying an activated excited-state pathway similar to hindered stilbenes.

**Fig. 1:**
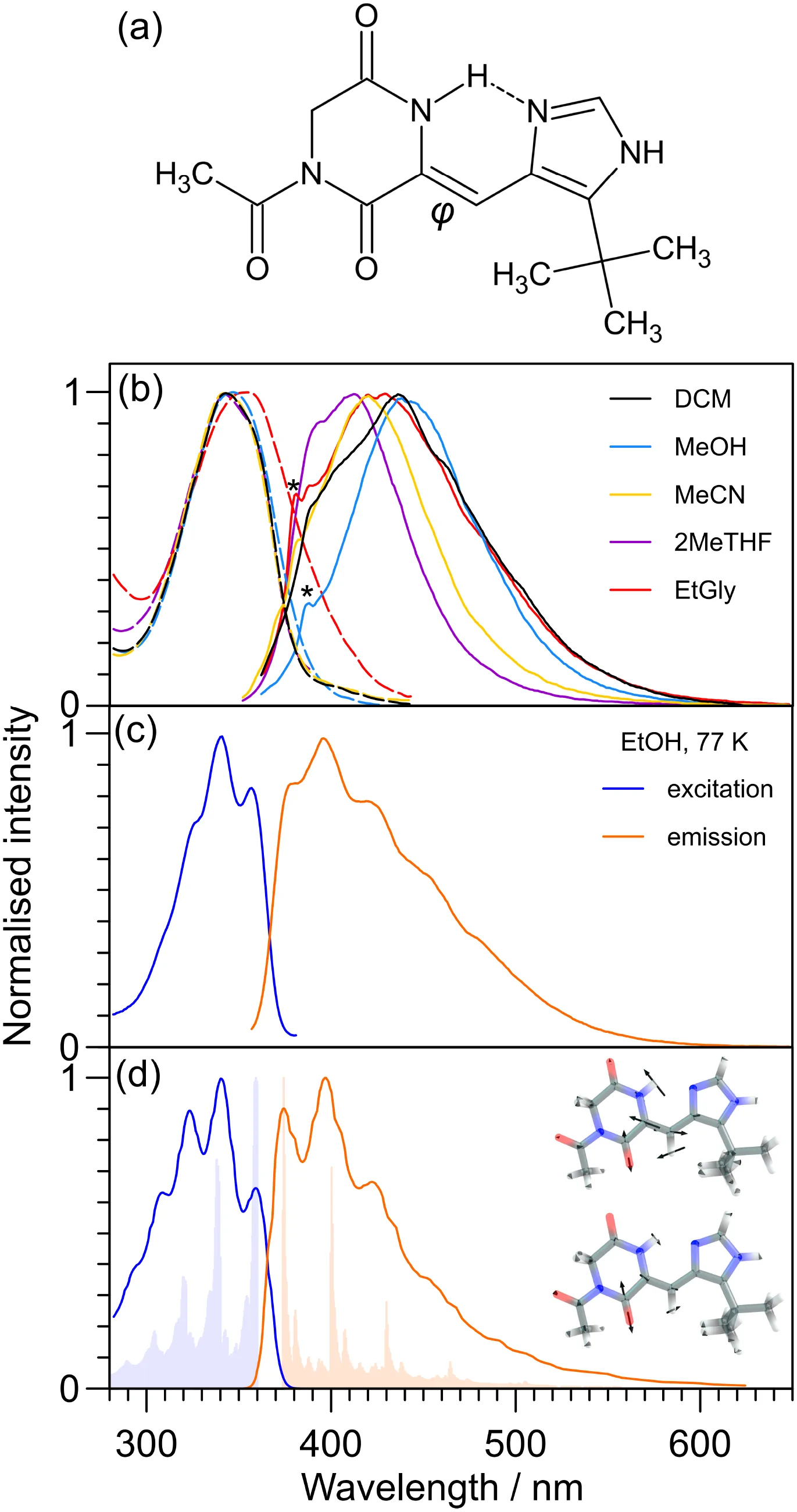
Spectrophotometry: (a) molecular structure of HP (note the intramolecular hydrogen bond straddling the isomerising double bond) and the acetate (protecting) group to avoid any potential complications from *lactam–lactim* tautomerisation, (b) absorption and fluorescence emission spectra (excited at 370 nm), (c) low-temperature (*T* ≈ 77 K) fluorescence excitation and emission spectra in ethanol, and (d) Franck–Condon simulations at *T* = 77 K of the S_0_–S_1_ (π π*) absorption and emission transitions using 400 cm^−1^ Gaussian peak convolutions. Stick spectra at *T* = 0 K (shaded) are shown. Major Franck–Condon active modes are shown.

HP absorbs over the 400–300 nm wavelength range (Fig. 1b, S_1_ ← S_0_ transition, see natural transition orbitals in the SI) in polar organic solvents, with a moderately large Stokes shift of 5000–6000 cm^−1^ (Table S1). Relative fluorescence quantum yields (*Φ*_F,rel_), measured against lumichrome, were ≈0.5% in methanol,≈0.8% in acetonitrile, and ≈2% in ethylene glycol. The larger *Φ*_F,rel_ in the more viscous ethylene glycol is consistent with frictional restriction of the large-amplitude torsional motion required for photoisomerisation and associated non-radiative decay, as widely observed for stilbene-like photoswitches.^8,9^ However, there is no simple correlation between either viscosity or polarity (in a Lippert–Mataga framework)^10^ and *Φ*_F,rel_ or Stokes shift across the solvents considered, indicating that bulk solvent properties alone do not define the condensed-phase photophysics of HP. Specific solute–solvent interactions, including hydrogen bonding, may therefore contribute to the accessibility of non-radiative relaxation pathways, as observed for other hydrogen-bonded alkene photoswitches.^11–13^

Flash freezing of HP in ethanol to *T* ≈ 77 K (forming a glassy matrix) narrowed the absorption and fluorescence emission bands to reveal vibronic structure (Fig. 1c). The vibronic structure was assigned through Franck–Condon simulations (Fig. 1d) performed using the time-dependent adiabatic-Hessian formalism with CAM-B3LYP/N07D assuming the harmonic oscillator approximation.^14,15^ In the absorption profile, the vibronic structure is dominated by an S_1_ state (harmonic) mode near 1688 cm^−1^, corresponding to a conjugated C–C/C=C stretching coordinate coupled appreciably to the C=O groups. Emission involves the S_0_ state mode near 1730 cm^−1^ (Fig. 1d, upper) and a mode near 1795 cm^−1^ (Fig. 1d, lower) with greater carbonyl-stretch character. In both spectra, lower-frequency skeletal and torsional modes (20–100 cm^−1^) contribute fine structure through combination bands. These lower frequency modes give rise to substantial hot band signal, broadening the envelope of the principal Franck–Condon modes, with increasing temperature.

The FTIR spectrum in DCM (Fig. 2a) shows two major bands centred at ≈1604 and ≈1693 cm^−1^. The higher-frequency band is assigned to the conjugated C–C/C=C stretching coordinate coupled to the C=O group identified in the Franck–Condon analysis, while the lower-frequency band is similarly delocalised but has substantially increased carbonyl stretch character (such calculated modes are strongly influenced by solvation).^17,18^ Anharmonic vibrational spectra were calculated at the B3LYP/N07D level (CAM-B3LYP for S_1_ state), a method that we have benchmarked against gas-phase IR spectra, typically giving mean absolute frequency errors of only a few wavenumbers.^15,17,19^ Selected TR-IR spectra (Fig. 2b) show a strong bleach centred at ≈1700 cm^−1^, and a small bleach at latter times near ≈1610 cm^−1^, consistent with the FTIR and calculated S_0_ state spectra. There are two pronounced excited-state absorption features centred at ≈1639 cm^−1^ and ≈1530 cm^−1^ assigned to corresponding S_1_ state vibrational modes based on agreement with the calculated S_1_ state IR spectrum (Fig. 2a). At long pump–probe delays (*e.g.* nanoseconds), the GSB feature does not fully refill and there is a persistent band at ≈1710 cm^−1^ consistent with predicted *E*-isomer IR spectrum (dotted black trace in Fig. 2a). The corresponding photoisomerisation quantum yield, *Φ*_I_ ≈ 25%, approximated from the main GSB band, is similar to values reported for other hemipiperazine photoswitches (*Φ*_I_ ≈ 15–16%).^3^

**Fig. 2:**
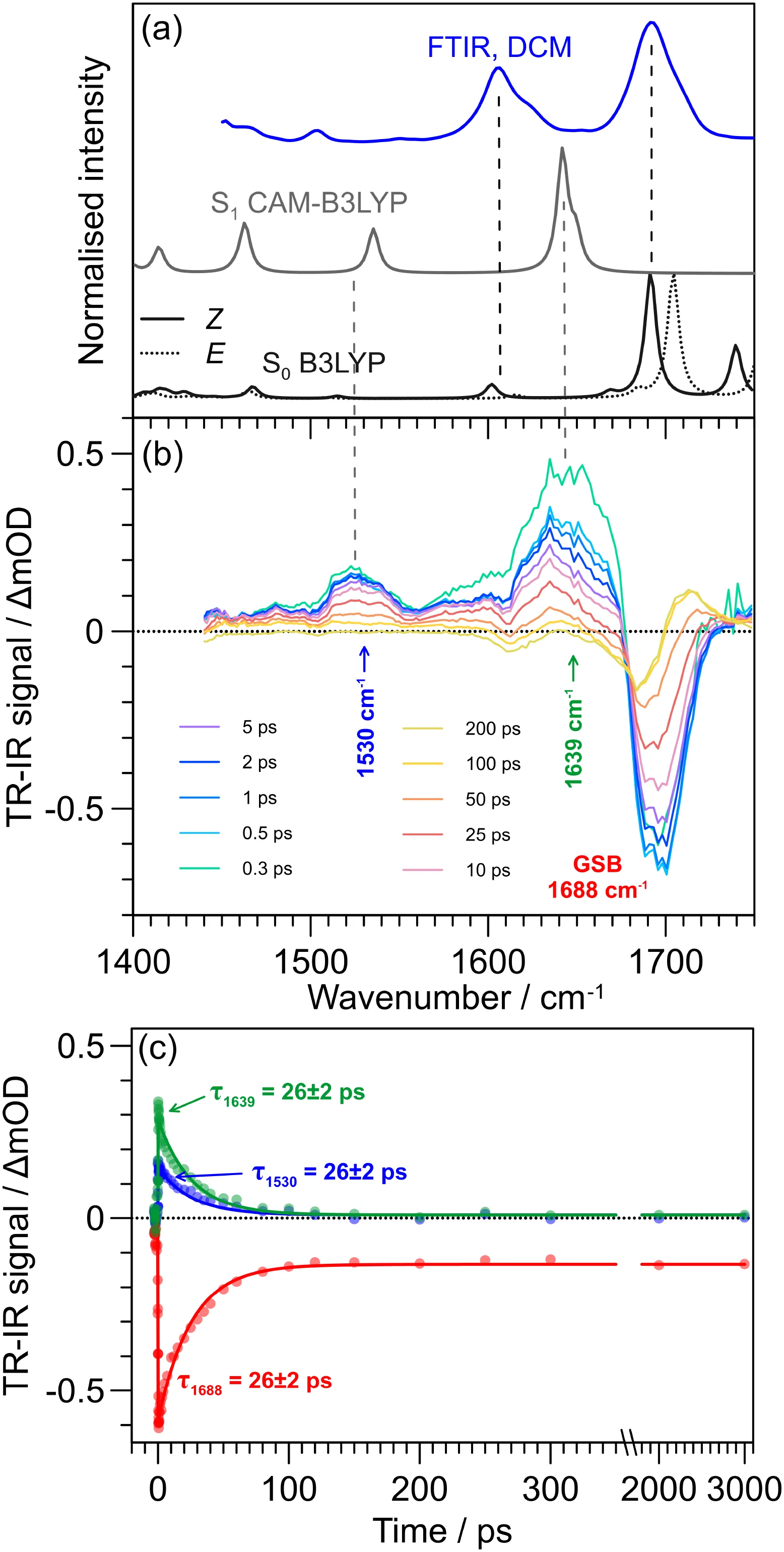
TR-IR spectroscopy of *Z*-HP in DCM: (a) FTIR spectroscopy (blue) and anharmonic (VPT2)^16^ calculated vibrational spectra for S_0_ (black) with frequency scaling factor 0.96 and S_1_ (grey) states. (b) Selected TR-IR spectra showing a strong ground state bleach (GSB) over 1680–1720 cm^−1^ range and two clear absorption bands centred at ≈1530 cm^−1^ and ≈1639 cm^−1^ that can be correlated with computed S_1_ state bands. (c) Fitted TR-IR kinetics using a mono-exponential decay. A persistent GSB after ≈100 ps with long-lived band centred at ≈1710 cm^−1^ correlating with the calculated *E* isomer IR spectrum. The small shoulder in the FTIR spectrum at ≈1705 cm^−1^ may be due to a trace amount of the *E* isomer formed during sample preparation.

Kinetics were extracted using two fit procedures with a single S_1_ → S_0_ decay lifetime: (i) integrated signals at three selected wavenumbers simultaneously fit to a Gaussian cross-correlation function convolved with a common exponential decay (Fig. 2c), and (ii) a global fit using GloTarAn^20^ (Fig. S1). Both fit procedures returned the same decay lifetime at *τ* = 26 ± 2 ps in DCM. In protic and hydrogen-bonding methanol-d4 (Fig. S2) with a similar viscosity to DCM, the excited-state decay is appreciably faster, at *τ* ≈ 16 ps.

The temperature dependence (*T* = 260–295 K) of *Φ*_F,rel_ (integrated intensity *I*_F_) was used to determine the apparent activation energy for non-radiative excited-state decay in an Arrhenius framework. The fluorescence quantum yield is given by *Φ*_F_ = *k*_r_/(*k*_r_ + *k*_nr_), where *k*_r_ and *k*_nr_ are the radiative and total non-radiative decay rates, respectively. Since *Φ*_F_ < 1%, *k*_nr_≫ *k*_r_, and hence *Φ*_F_ ≈ *k*_r_/*k*_nr_. For measurements on pure *Z*-isomer solution at different temperatures over a limited range, we take *I*_F_ ∝ *Φ*_F_. Assuming that *k*_r_ and the experimental proportionality factor are temperature independent, then *k*_nr_ ∝ *I*_F_^−1^. Thus, fitting ln(*k*_nr_) against *T*^−1^, according to *l*n(*k*_nr_) = *C* − *E*_a_/*RT* (Fig. 3a) provided the apparent activation energies of 15 ± 1 kJ mol^−1^ in DCM,9.5 ± 0.9 kJ mol^−1^ in methanol and 10.8 ± 0.3 kJ mol^−1^ in methanol-d4. These values are regarded as apparent activation energies for the overall non-radiative excited-state decay rather than as direct measurements of a single microscopic photoisomerisation barrier (free-energy barrier). Nevertheless, the solvent dependence of the apparent activation energies is qualitatively consistent with the TR-IR lifetimes, with the higher barrier in DCM accompanying the slower S_1_ state decay, but no obvious deuterium isotope effect in the protic solvent. Quantitative agreement is not inherently expected because the effective Arrhenius prefactor may be solvent dependent. For example, for *E*-stilbene at *T* = 293 K, the excited-state lifetime decreases from ≈89 ps in *n*-hexane to ≈40 ps in acetonitrile, a 2.2-fold decrease, while the apparent barrier decreases from ≈18.2 to ≈15.3 kJ mol^−1^ and the fitted prefactor decreases from 19.4 to 13.1 ps^−1^.^21^ Thus, considering just the barrier difference overestimates the observed change in isomerisation rate. The solvent dependence of *E*-stilbene is associated with increasing polar character on twisting, which is preferentially stabilised in polar solvents^22^ (while planar *E*-stilbene is nonpolar, there is a calculated dipole moment of ≈8.0 D at the twisted-pyramidalised S_1_ state minimum).^23^ For HP, the two-fold faster decay in methanol-d4 compared with DCM, combined with Δ*E*_a_ ≈ 4 kJ mol^−1^, would require an effective Arrhenius prefactor three-fold smaller in methanol-d4. Gas-phase calculations (described next) suggest that the molecular dipole moment in HP changes only slightly from 9.30 D at the optimised S_0_ state geometry to 9.54 D at the planar S_1_ state minimum, but increases to 12.36 D at the S_1_ state barrier geometry and reaches 19.4 D in the twisted region beyond the barrier (similar changes found with CAM-B3LYP in MRSF-TDDFT). This substantial increase in electronic polarisation along the isomerisation coordinate suggests that preferential solvent stabilisation of the more polar barrier region may contribute to the lower apparent activation energy in methanol-d4, while the substantial coupling to solvent reorganisation in the twisted region may influence the effective Arrhenius prefactor.

**Fig. 3:**
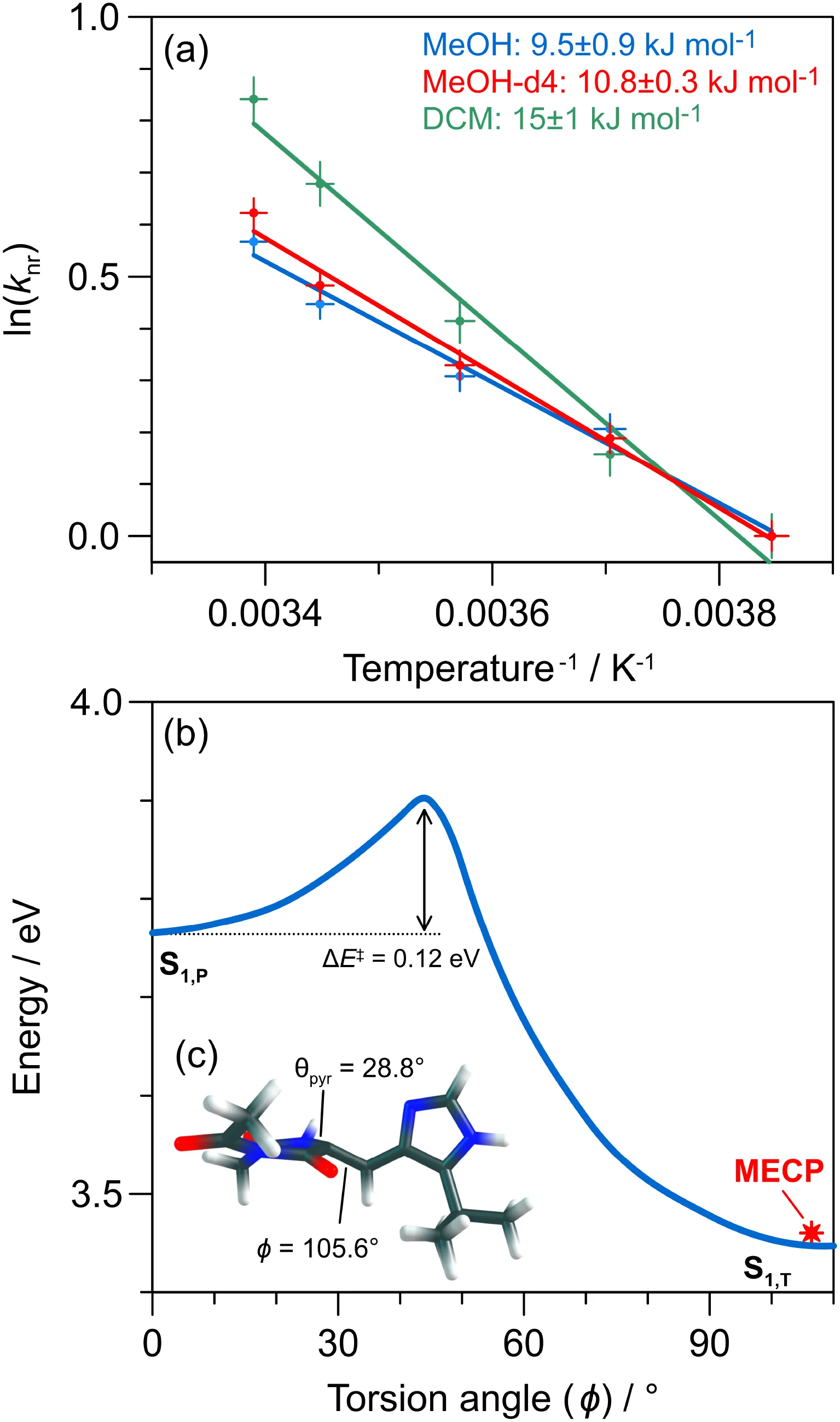
Excited-state activation energy determination for *Z*-HP: (a) Arrhenius Law analysis. For each solvent, integrated fluorescence intensities were normalised to the value at the lowest temperature (260 K) prior to conversion to the relative non-radiative rate proxy, hence ln(*k*) = 0 at *T* = 260 K. (b) MRSF-TDDFT S_1_ relaxed state potential energy surface for torsion about *ϕ*, where *ϕ* = 0° is defined as the *Z* isomer. (c) Illustration of the MECP.

The molecular origin of activated decay was examined using gas-phase potential-energy surface calculations with MRSF-TDDFT.^24^ The calculated S_1_ state surface (Fig. 3b) contains a shallow planar minimum separated from the S_1_/S_0_ crossing region by a barrier of 0.12 eV (11.6 kJ mol^−1^), which lies within the 9.5–15 kJ mol^−1^ range determined above. The agreement is notable given that the calculated value is a gas-phase electronic energy barrier whereas the experimental values are solvent-dependent apparent activation energies. The minimum energy crossing point (MECP) on the S_1_/S_0_ conical intersection seam is reached through torsion about the photoisomerising C=C bond to ≈105.6°, substantial pyramidalisation at the sp^2^ carbon of the diketopiperazine moiety (*θ*_pyr_ = 28.8°), and puckering of the adjacent six-membered ring (Fig. 3c). Importantly, the MECP lies in the minimum energy region of the twisted S_1_ state surface, with no distinct twisted S_1_ state minimum separating it from the crossing seam (no unassigned transients),^17,18^ in contrast to some hydrazone photoswitches where ultrafast spectroscopy and calculations identify a distinct twisted S_1_ state minimum close to the isomerising conical intersection.^25^ The combined results therefore support activated escape from a shallow planar S_1_ state minimum (S_1,P_) followed by direct access to a low-lying region of the isomerising conical-intersection seam, providing an efficient non-radiative decay pathway consistent with the substantial *Φ*_F_.^26–28^

The solvent effect on the excited-state lifetime of HP is more modest than that observed for several related photoswitches with intramolecular bridging hydrogen bonds in which specific solute–solvent interactions strongly influence the photoisomerisation coordinate. The HP lifetime decreases from *τ* = 26 ± 2 ps in DCM to ≈16 ps in methanol-d4, accompanied by the decrease in apparent activation energy described above. For comparison, the S_1_ state lifetime of the hemiindigo *Z*-DABI decreases from 76 ps in toluene to 9.3 ps in methanol, an approximately eight-fold acceleration attributed to stronger intermolecular hydrogen bonding in the excited state.^13^ Hemiindigos similarly show pronounced changes in fluorescence lifetime and photoisomerisation yield upon modification of the hydrogen-bonding framework,^29^ while multireference calculations on related hemithioindigos show that proton-transfer and isomerisation coordinates can become strongly coupled on the excited-state surface.^30^ An N–H⋯O=C intramolecular hydrogen-bonded hydrazone similarly shows excited-state recovery on ≈140 ps and ≈34 ps timescales in toluene and methanol, respectively, attributed to competition between intramolecular and solute–solvent hydrogen bonding.^12^ Other ultrafast studies of substituted hydrazones likewise report picosecond structural relaxation followed predominantly by internal conversion rather than photoisomerisation in some solvents.^31^

Given the intramolecular hydrogen bond in HP, excited-state proton transfer represents a possible competing relaxation pathway that, if active, should be clearly discerned by TR-IR spectroscopy. Because proton transfer changes the local bonding, it results in strongly shifted or new vibrational bands characteristic of the new tautomer. For example, formation of the proton-transferred tautomer of 2-(2′ -hydroxyphenyl)benzothiazole produces new N–H and C=O stretching bands,^32^ while ultrafast TR-IR spectroscopy of 3-hydroxyflavone directly resolved vibrational bands of the *keto* tautomer formed within <100 fs.^33^ For HP, however, the observed excited-state spectrum is well reproduced by calculations of the di-*keto* S_1_ state structure. Therefore, there is no spectroscopic evidence for appreciable accumulation of an excited-state intramolecular proton transfer tautomer of HP in DCM. Similarly, the TR-IR spectroscopy in methanol-d4 (Fig. S2), a hydrogen-bonding and protic solvent, shows no additional bands that could be assigned to a proton transfer species.

The mechanistic picture established for *Z* → *E* HP as an ultrafast, substantial isomerisation quantum yield (*Φ*_I_ ≈ 25%) photoswitch provides a basis for understanding how structural modification of the hemipiperazine scaffold may tune the competition between fluorescence and photoisomerisation. Changes to arylidene substitution, conformational rigidity, and intramolecular hydrogen-bonding geometry are expected to alter access to the S_1_/S_0_ state crossing seam across this growing family of photoswitches.^5,7^ Related strategies utilising ultrafast spectroscopy and dynamics to guide rational design have proven effective in other alkene and imine photoswitch development, where donor–acceptor substitution, structural rigidity, and intramolecular hydrogen bonding can alter the excited-state lifetime and the competition between fluorescence, internal conversion, and photoisomerisation.^30,31,34–36^

## Conflicts of interest

There are no conflicts to declare.

## Supplementary Material

CC-062-D6CC05789F-s001

## Data Availability

The data that support the findings of this study are available from the corresponding author upon reasonable request. Supplementary information (SI): experimental and computational methods, TR-IR data and analysis, natural transition orbitals. See DOI: https://doi.org/10.1039/d6cc05789f.
