# Supplementary material for: Activated excited-state isomerisation in a hemipiperazine photoswitch
Source: Chem Commun (Camb). 2026 Sep 28;62(83):20973–7. doi: 10.1039/d6cc05789f (PMC13624959; doi:10.1039/d6cc05789f)
Supplement: Supplementary file 1 [file CC-062-D6CC05789F-s001.pdf]

Supplementary Information (SI) for ChemComm.
This journal is © The Royal Society of Chemistry 2026

Electronic Supplementary Information for

Activated excited-state isomerisation in a hemipiperazine photoswitch

Eleanor K. Ashworth, Anam Fatima, Samuel Bleckmann, Zbigniew L. Pianowski, Joseph A. Wright,
Stephen R. Meech, Partha Malakar, and James N. Bull

Contents

1 Sample and solvents **S3**

2 Methods **S3**

2.1 Spectrophotometry S3
2.2 Computational S3
2.3 TR-IR S3

3 Spectrophotometry **S4**

4 EADS in DCM **S5**

5 TR-IR in methanol-d4 **S6**

6 Photostationary state **S7**

7 NTOs **S8**

8 Critical point geometries in Å **S9**

8.1 S_0 state geometry S9
8.2 S_1 planar state ($S_{1,P}$) geometry S10
8.3 S_0/S_1 MECP geometry S11

1 Sample and solvents

HP was synthesised following published procedures and confirmed by ^{1}H-NMR spectroscopy.[1] Solvents used for spectroscopic analysis were:

H_2O	Obtained from in-house Veolia PURELAB flex pure water system
H_2O-d2	Goss Scientific, 99.8 atom % D
methanol	Fisher, ≥99.9%
methanol-d4	Thermo Scientific, 99.8 atom % D
ethanol	Fisher, ≥99.8%
MeCN	Fisher, ≥99.8%
DCM	Fisher, ≥99%
2MeTHF	Acros Organics, 99+%
EtGly	Sigma-Aldrich, ≥99%

MeCN is acetonitrile, DCM is dichloromethane, 2MeTHF is 2-methyl-tetrahydrofuran, and EtGly is ethylene glycol.

2 Methods

2.1 Spectrophotometry

Room temperature absorption and excitation/emission spectra were recorded with a Perkin-Elmer Lambda XLS spectrometer and an Edinburgh Instruments FS5 spectrofluorometer (SC-05 module), respectively. $T \approx 77$ K excitation/emission spectra were recorded using the Edinburgh Instruments FS5 spectrofluorometer (SC-70 module). The fluorescence quantum yield of HP is very low, so accurate measurement of absolute quantum yield was not possible with an integrating sphere. Instead, relative quantum yield was determined against a standard solution of lumichrome whose quantum yield is well known.[2,3]

Temperature-dependent emission spectra were recorded (over the T=260–295 K range) using the Edinburgh Instruments FS5 spectrofluorometer with the SC-80 module and an Oxford Instruments cryostat. Measurements were performed using a fresh sample (from a bulk solution at a constant concentration) for each temperature to avoid photoisomerisation by the probe light.

2.2 Computational

The N07D basis set considered in this work takes 6-31G and adds selected dispersion and polarisation functions, which improves the accuracy of calculated vibrational frequencies for large aromatic and highly conjugated molecules.[4] S_1 state optimised geometries and frequencies were computed at the CAM-B3LYP/N07D level of theory.[5] These computations were performed using tight optimisation criteria (1×10^{-5}) and an ultrafine integration grid with Gaussian 16.[6] MRSF-TDDFT calculations were performed at the BH&HLYP/cc-pVDZ level of theory.[7,8]

Mixed-reference spin-flip time-dependent density functional theory (MRSF-TDDFT) calculations of the potential energy surface and the MECP were performed in OpenQP 1.2.[9] The BH&HLYP functional was employed because the formulation of MRSF-TDDFT couples spin-flip configurations primarily through exact exchange, favouring hybrid functionals with a large Hartree-Fock exchange contribution. BH&HLYP, containing 50% exact exchange, has consequently become a standard choice for MRSF-TDDFT calculations.[10,11]

2.3 TR-IR

Time-resolved infrared (TR-IR) spectroscopy on HP at a concentration of 1–2 mM in dichloromethane (DCM) and methanol-d4 was performed on the ULTRA LifeTime system at the Central Laser Facility, Research Complex at Harwell, Rutherford Appleton Laboratory.[12] The dissolved sample, loaded in a 50 μm pathlength CaF_2 cell, was excited with 340 nm light (≈500 nJ pulse^{-1}, 150 μm spot size, 1 kHz repetition rate), followed by a delayed IR probe (cross correlation ≈200 fs) to capture the transient IR spectrum. The sample was rastered and flowed to minimise photodegradation, with pump-on/pump-off measurements acquired under magic-angle polarisation. Spectral calibration used a standard polystyrene IR spectrum. FTIR spectra were recorded using a Bruker Vertex 80 equipped with a CaF_2 liquid cell. The 1450–1750 cm^{-1} spectral window considered here, defined by solvent IR transparency, encompasses vibrations with substantial C=C and C=O character and therefore probes vibrational modes most directly coupled to structural changes along the photoisomerisation coordinate.

3 Spectrophotometry

Table S1 Spectroscopic properties of *Z*-HP at $T = 291$ K (λ in nm and Stokes shift, $\tilde{\nu}$, in cm^{-1}). Emission spectra recorded exciting at λ_{abs}. Uncertainties: $\lambda_{abs} \pm 2$ nm, $\lambda_{em} \pm 2$ nm, $\tilde{\nu} \pm 400\,cm^{-1}$. ε is solvent polarity (dielectric constant), and η is solvent viscosity in cP.

	λ_{abs}	λ_{em}	$\tilde{\nu}$	ε	η
DCM	345	435	5997	8.93	0.413
MeOH	346	440	6174	33.0	0.544
MeCN	341	419	5459	36.64	0.369
2MeTHF	341	414	5171	6.97	0.4
EtGly	347	420	5009	41.1	16.1
H_2O [a]	341	417	5345	80.1	0.89
EtOH [b]	345	407	4415	—	—

[a] H_2O is ≈99% H_2O + ≈1% CH_3OH to improve solubility. ε and η values from CRC Handbook (η for 2MeTHF from Fisher SDS). [b] $T \approx 77$ K excitation and emission.

Neither the absorption nor emission maxima (in terms of energy) show a systematic dependence on solvent polarity. The absorption maximum is comparatively insensitive to solvent, varying only from 341 to 347 nm across the room-temperature solvent series, whereas the emission maximum shows a larger variation from 414 to 440 nm. Importantly, however, the latter variation is not monotonic with solvent polarity. Thus, examination of the individual contributions to the Stokes shift does not reveal a correlation that is obscured when considering the Stokes shift alone. Similarly, no systematic dependence of the emission energy on viscosity was found.

It is worth noting that our calculations indicate that the S_2 (vertical $E = 5.49$ eV, $f = 0.0005$) and S_3 (vertical $E = 5.65$ eV, $f = 0.04$) states lie much higher in energy that the bright ($f = 0.84$) S_1 state, consistent with the UV-vis spectrophotometry, particularly at low temperature ($T \approx 77$ K), showing a single band that is well-described the the Franck-Condon simulation.

4 EADS in DCM

Evolution associated decay spectra (EADS) for TR-IR data of *Z*-HP in DCM obtained from a GloTarAn global fit are shown in Fig. S1. XEADS1 corresponds to the initial transient spectrum, while XEADS2 corresponds to the final, persistent spectrum. The positive absorption bands in XEADS2 are attributed to the *E* isomer.

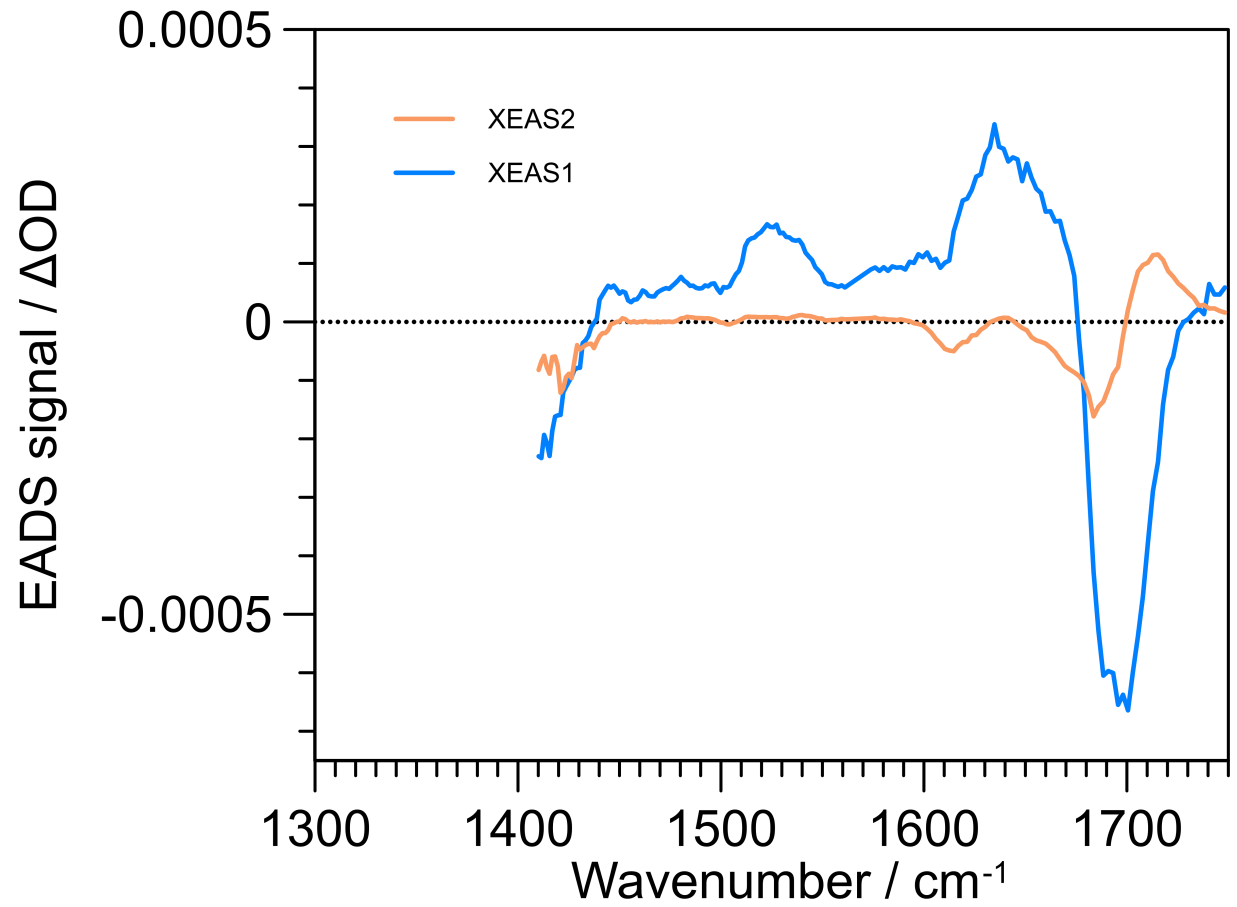

Fig. S1 EADS from global fit of TR-IR data of HP in DCM.

5 TR-IR in methanol-d4

TR-IR spectra and associated kinetics of HP in methanol-d4 are shown in Fig. S2. As in DCM, prominent ground state bleach (GSB) features are observed around 1700 cm^{-1}, broadly consistent with the calculated gas-phase modes just below 1700 cm^{-1} and at ≈1740 cm^{-1}. Differences between the calculated and experimental frequencies are expected because hydrogen-bonding interactions with the solvent can appreciably shift these modes. Notably, no transient absorption bands exhibit a delayed rise over the first few picoseconds followed by subsequent decay, as would be expected for accumulation of an excited state (proton transferred) tautomer with a different vibrational spectrum. Thus, there is no spectroscopic evidence for appreciable population of such an intermediate. As in DCM, the GSB does not fully recover at long pump-probe delays (e.g. for <200 ps), consistent with formation of the *E* isomer.

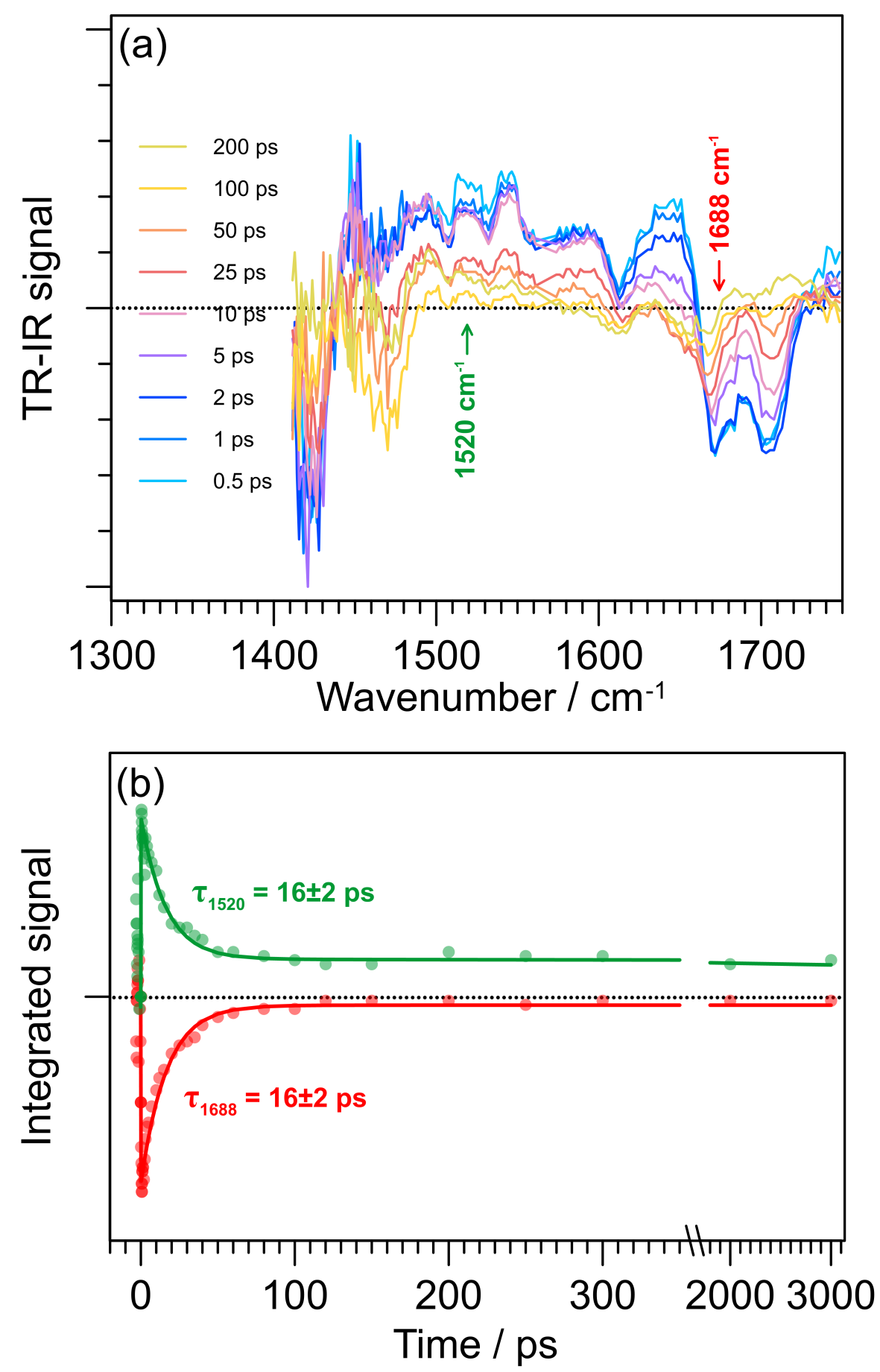

Fig. S2 TR-IR of HP in methanol-d4: (a) Selected TR-IR spectra, (b) fits of integrated bands with a single exponential decay.

6 Photostationary state

The dynamics reported in the manuscript focussed on the more stable *Z* isomer exhibiting an intramolecular hydrogen bond. Irradiation of HP in solution with 365 nm light (ThorLabs M365L3 LED) formed a photostationary state (PSS) containing both *Z* and *E* isomers (Fig. S3b), where the latter does not support an intramolecular hydrogen bond. The PSS spectrum reveals an absorption band centred at ≈400 nm due to the *E* isomer. Future experiments might like to perform pump-probe spectroscopy on the PSS at 400 nm to characterise the excited-state dynamics of the *E* isomer as the *Z* isomer.

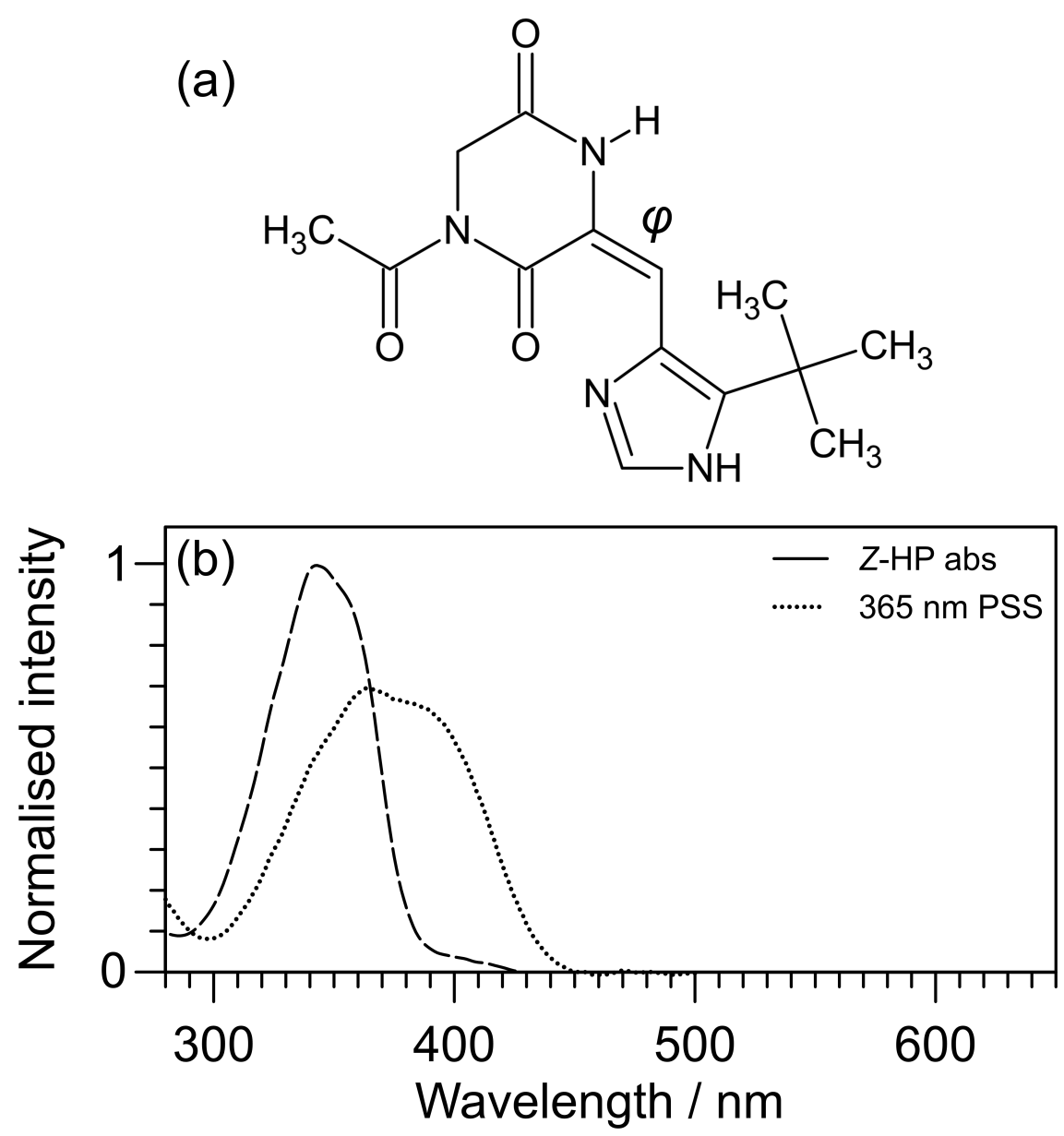

Fig. S3 *E*-HP: (a) molecular structure showing no intramolecualar hydrogen bond across the isomerising C=C bond. (b) absorption spectrum of *Z* and the photostationary state (PSS) containing the *E* isomer.

7 NTOs

Natural transition orbitals (NTOs) associated with the $S_1 \leftarrow S_0$ transition are shown in Fig. S4. The transition involves localised $\pi\pi^*$ character over the isomerising double bond.

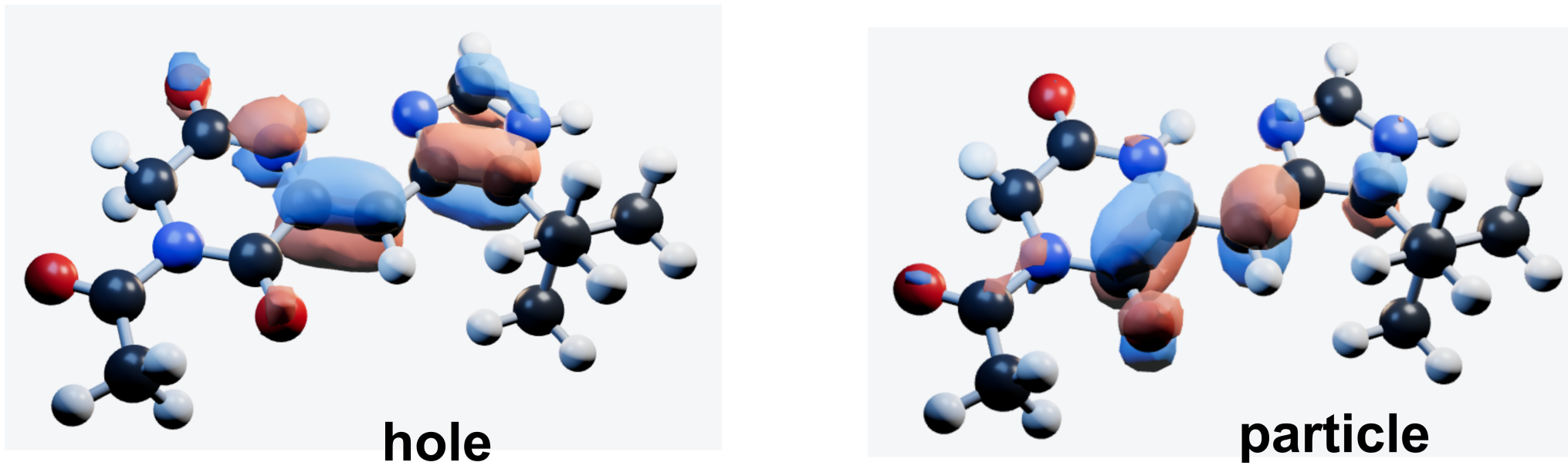

Fig. S4 Natural transition orbitals for the Z isomer of HP.

8 Critical point geometries in Å

8.1 S_0 state geometry

Atom	*x*	*y*	*z*
C	-1.064859147100	1.191139483000	-1.306188521100
C	-0.282641329200	-1.385310636300	-0.199677026200
C	0.667057118000	-0.262991286700	-0.412485695800
O	-1.407716001300	2.251459829600	-1.767268244500
N	-1.602337804100	-1.166123398000	-0.554742127000
O	0.100584370100	-2.434501445300	0.264152317900
N	0.211065119500	0.927986813900	-0.947526166800
H	0.904403453100	1.659149421100	-1.074060046700
C	1.955493077400	-0.471654080300	-0.063093037100
C	3.036925284800	0.475898855400	-0.180953618100
C	4.366166454000	0.313577656400	0.168585483000
N	2.840081668600	1.742579597200	-0.694263490600
C	3.996339775200	2.330021956500	-0.660798893200
H	4.206634745800	3.336783407900	-0.989398836600
N	4.946007995300	1.513121489500	-0.150482704500
H	5.912681289300	1.745849264300	-0.028275071600
C	5.146024674000	-0.837587752400	0.768136322400
C	6.615958010300	-0.448246692700	0.952066324800
H	7.167694885000	-1.287021621600	1.382605352100
H	7.095381883900	-0.202297522000	-0.001277442100
H	6.730706734200	0.399202508200	1.635807623300
C	4.566616749000	-1.200203101600	2.142929858000
H	4.623596212100	-0.349016129600	2.827013853600
H	3.520966831100	-1.504926737600	2.075350522800
H	5.130370146200	-2.029494651700	2.581232576200
C	5.080272142800	-2.054621787800	-0.164962795500
H	4.053831494900	-2.389894903200	-0.323398898800
H	5.509426084100	-1.821015471000	-1.143373754900
H	5.643347513600	-2.888531781600	0.265163755900
H	2.162836475200	-1.450781009900	0.339480489100
C	-2.628821239200	-2.126547711700	-0.413729706700
C	-2.342060196700	-3.488172604000	0.143367990100
H	-1.596863785300	-4.011009796900	-0.455413378500
H	-1.932004902100	-3.421795592700	1.150728777800
H	-3.288543737500	-4.026891825300	0.143970125600
O	-3.736947835500	-1.807423003200	-0.756247708400
C	-2.080313450600	0.099270183700	-1.110905902100
H	-2.870631773600	0.501349285200	-0.475046023000
H	-2.549921129600	-0.082962601200	-2.078695108400

8.2 S_1 planar state ($S_{1,P}$) geometry

Atom	*x*	*y*	*z*
C	-1.066040503400	1.171449451600	-1.303781566900
C	-0.289212714000	-1.378670218200	-0.191315795800
C	0.633792501900	-0.294781248200	-0.409615461000
O	-1.350973724900	2.247399738400	-1.764890237400
N	-1.632024725900	-1.167159882900	-0.557303598800
O	0.111515806700	-2.442532245000	0.295041305300
N	0.222505411200	0.883962305900	-0.939412685200
H	0.926147917600	1.610632249600	-1.067423082500
C	1.989622856900	-0.489475312800	-0.054586501100
C	3.016577290600	0.453989763900	-0.179216842400
C	4.392206760700	0.319043731400	0.171822097700
N	2.817794462300	1.727911096500	-0.694074432000
C	3.967999203700	2.325629800200	-0.663717822500
H	4.170413416700	3.333840760400	-0.992743164700
N	4.936710991300	1.517124102100	-0.152581503600
H	5.900848423600	1.766886899300	-0.034255704300
C	5.174419247000	-0.818107175400	0.766772002700
C	6.642305435900	-0.424683603800	0.948827051700
H	7.195875781900	-1.262308751200	1.378484160400
H	7.120594265000	-0.178177033200	-0.005112611600
H	6.755721774700	0.422286446800	1.633618933300
C	4.588388643100	-1.182352471100	2.142500151000
H	4.674406720000	-0.342047506700	2.836756447400
H	3.534828657900	-1.456919670500	2.074787401500
H	5.134069666000	-2.032066538500	2.563375564100
C	5.103354567400	-2.037839061700	-0.168565218600
H	4.074404471900	-2.354617616500	-0.344878182300
H	5.557165893600	-1.813164400800	-1.137771974400
H	5.644938949600	-2.877224968400	0.277329176500
H	2.196038427100	-1.467141609600	0.348366372200
C	-2.651814181300	-2.105672853200	-0.429843299300
C	-2.369884072800	-3.475944803400	0.113683135700
H	-1.617418130800	-3.990404427200	-0.483015504600
H	-1.965981800400	-3.421117510900	1.123979615900
H	-3.315937849600	-4.015826046600	0.100339318600
O	-3.768554166300	-1.781506217500	-0.771084573500
C	-2.097785081400	0.101274815900	-1.102005945200
H	-2.878145251200	0.522638553200	-0.462268583300
H	-2.585206598500	-0.062444095600	-2.066230299100

8.3 S_0/S_1 MECP geometry

Atom	*x*	*y*	*z*
C	-1.279341267300	0.989425547700	-1.441882647900
C	-0.158651496900	-1.241045016200	-0.030670042200
C	0.338585476800	0.023507354700	0.066792607900
O	-1.928421936500	1.924881205600	-1.846350175100
N	-1.410111069800	-1.356367050000	-0.697423427500
O	0.530373590900	-2.241186289200	0.317234080300
N	-0.220862128600	1.143617670900	-0.593814898000
H	-0.227775362200	2.036417269500	-0.125574865300
C	1.771795540000	-0.097300813700	0.276820406600
C	2.439094090000	0.077388534400	1.460289359300
C	3.795799084800	-0.291113607800	1.735078147300
N	1.854089630800	0.581046399100	2.620862081900
C	2.760704552700	0.537947311900	3.513363603100
H	2.678409606900	0.863478222300	4.540177814800
N	3.953220290800	0.004393674800	3.018306572000
H	4.792301578600	-0.151776900700	3.549142424400
C	4.858094472400	-0.974367938200	0.910529045700
C	6.150841455600	-1.116804884800	1.719530096700
H	6.902190508300	-1.621743136200	1.109828349600
H	6.565635004900	-0.144980132200	2.006567852000
H	6.010797357900	-1.727312642400	2.617459654500
C	4.342239471300	-2.377154976800	0.538205524200
H	4.212408392400	-2.990916859100	1.433644588400
H	3.378968786000	-2.349599314900	0.027564116900
H	5.076130485900	-2.867688349600	-0.106475040300
C	5.168128042900	-0.149064915700	-0.347367809600
H	4.314799242900	-0.070205446000	-1.020464214000
H	5.492398837200	0.862758254700	-0.088922665000
H	5.978225797100	-0.632872399600	-0.898253500200
H	2.406744606500	-0.386140579700	-0.562901528300
C	-2.332355709300	-2.357702414300	-0.500910900800
C	-2.130942158600	-3.251496265800	0.691524906600
H	-1.214851631100	-3.829795243200	0.575071254500
H	-2.012784142100	-2.659740678400	1.602444085300
H	-3.002754773000	-3.899175502100	0.771516657600
O	-3.278010304600	-2.487341389300	-1.245363823000
C	-1.586732866400	-0.448152323900	-1.818781535400
H	-2.618109394800	-0.497776196000	-2.153138851200
H	-0.943321802300	-0.738324279700	-2.660998845800

References

1 S. Kirchner, A.-L. Leistner, P. Gödtel, A. Seliwjorstow, S. Weber, J. Karcher, M. Nieger and Z. Pianowski, *Nat. Comm.*, 2022, **13**, 6066.

2 E. Sikorska, I. V. Khmelinskii, W. Prukała, S. L. Williams, M. Patel, D. R. Worrall, J. L. Bourdelande, J. Koput and M. Sikorski, *J. Phys. Chem. A*, 2004, **108**, 1501–1508.

3 B. Maity, A. Chatterjee and D. Seth, *Chemical Physics Letters*, 2013, **565**, 108–115.

4 V. Barone, M. Biczysko and J. Bloino, *Phys. Chem. Chem. Phys.*, 2014, **16**, 1759–1787.

5 T. Yanai, D. P. Tew and N. C. Handy, *Chem. Phys. Lett.*, 2004, **393**, 51–57.

6 M. J. Frisch, G. W. Trucks, H. B. Schlegel, G. E. Scuseria, M. A. Robb, J. R. Cheeseman, G. Scalmani, V. Barone, G. A. Petersson, H. Nakatsuji, X. Li, M. Caricato, A. V. Marenich, J. Bloino, B. G. Janesko, R. Gomperts, B. Mennucci, H. P. Hratchian, J. V. Ortiz, A. F. Izmaylov, J. L. Sonnenberg, D. Williams-Young, F. Ding, F. Lipparini, F. Egidi, J. Goings, B. Peng, A. Petrone, T. Henderson, D. Ranasinghe, V. G. Zakrzewski, J. Gao, N. Rega, G. Zheng, W. Liang, M. Hada, M. Ehara, K. Toyota, R. Fukuda, J. Hasegawa, M. Ishida, T. Nakajima, Y. Honda, O. Kitao, H. Nakai, T. Vreven, K. Throssell, J. A. M. Jr., J. E. Peralta, F. Ogliaro, M. J. Bearpark, J. J. Heyd, E. N. Brothers, K. N. Kudin, V. N. Staroverov, T. A. Keith, R. Kobayashi, J. Normand, K. Raghavachari, A. P. Rendell, J. C. Burant, S. S. Iyengar, J. Tomasi, M. Cossi, J. M. Millam, M. Klene, C. Adamo, R. Cammi, J. W. Ochterski, R. L. Martin, K. Morokuma, O. Farkas, J. B. Foresman and D. J. Fox, *Gaussian 16 Revision B.01*, 2016, Gaussian Inc. Wallingford CT.

7 A. D. Becke, *J. Chem. Phys.*, 1993, **98**, 1372–1377.

8 T. H. Dunning, *J. Chem. Phys.*, 1989, **90**, 1007–1023.

9 V. Mironov, K. Komarov, J. Li, I. Gerasimov, H. Nakata, M. Mazaherifar, K. Ishimura, W. Park, A. Lashkaripour, M. Oh, M. Huix-Rotllant, S. Lee and C. H. Choi, *J. Chem. Theo. Comput.*, 2024, **20**, 9464–9477.

10 W. Park, K. Komarov, S. Lee and C. H. Choi, *J. Phys. Chem. Lett.*, 2023, **14**, 8896–8908.

11 S. Lee, W. Park and C. H. Choi, *Acc. Chem. Res.*, 2025, **58**, 208–217.

12 G. M. Greetham, P. Burgos, Q. Cao, I. P. Clark, P. S. Codd, R. C. Farrow, M. W. George, M. Kogimtzis, P. Matousek, A. W. Parker, M. R. Pollard, D. A. Robinson, Z.-J. Xin and M. Towrie, *Appl. Spec.*, 2010, **64**, 1311–1319.
